# Evolution and functional divergence of the anoctamin family of membrane proteins

**DOI:** 10.1186/1471-2148-10-319

**Published:** 2010-10-21

**Authors:** Vladimir M Milenkovic, Marisa Brockmann, Heidi Stöhr, Bernhard HF Weber, Olaf Strauss

**Affiliations:** 1 Experimental Ophthalmology, Regensburg University Medical Center, 93053 Regensburg, Germany; 2 Institute of Human Genetics, University of Regensburg, Franz-Josef-Strauss-Allee 11, 93053 Regensburg, Germany

## Abstract

**Background:**

The anoctamin family of transmembrane proteins are found in all eukaryotes and consists of 10 members in vertebrates. Ano1 and ano2 were observed to have Ca^2+ ^activated Cl^- ^channel activity. Recent findings however have revealed that ano6, and ano7 can also produce chloride currents, although with different properties. In contrast, ano9 and ano10 suppress baseline Cl^- ^conductance when co-expressed with ano1 thus suggesting that different anoctamins can interfere with each other. In order to elucidate intrinsic functional diversity, and underlying evolutionary mechanism among anoctamins, we performed comprehensive bioinformatics analysis of anoctamin gene family.

**Results:**

Our results show that anoctamin protein paralogs evolved from several gene duplication events followed by functional divergence of vertebrate anoctamins. Most of the amino acid replacements responsible for the functional divergence were fixed by adaptive evolution and this seem to be a common pattern in anoctamin gene family evolution. Strong purifying selection and the loss of many gene duplication products indicate rigid structure-function relationships among anoctamins.

**Conclusions:**

Our study suggests that anoctamins have evolved by series of duplication events, and that they are constrained by purifying selection. In addition we identified a number of protein domains, and amino acid residues which contribute to predicted functional divergence. Hopefully, this work will facilitate future functional characterization of the anoctamin membrane protein family.

## Background

The anoctamin (ano, also known as TMEM16) proteins represent a novel family of membrane proteins with 10 members (ano1-10) in mammals [1-11]. Some members are over-expressed in various cancers and diseases [12-18]. Anoctamins are highly hydrophobic proteins with eight transmembrane domains (TMD) and one re-entry loop [19]. Anoctamin proteins have tissue-specific patterns of expression [20,21]. Although electrophysiological and biochemical studies in both native and heterologous expression systems provided important clues to understanding the function of anoctamin membrane proteins, the biological roles have been elucidated for only a few members of this family [2-6,21-24]. Ano1 functions as a Ca^2+^-activated Cl^- ^channel in a broad range of tissues, and it can be activated by cell swelling [22]. Ano2 expression is confined to the photoreceptor synaptic terminals in retina and the olfactory sensory neurons where it functions as a Ca^2+^-activated Cl^- ^channel [3,4]. Ano6 and ano7 can also induce Cl^- ^conductance when over expressed in FRT cells [21], although the function of these proteins is not clear. However, it seems that not all anoctamin proteins operate as Ca^2+^-activated Cl^- ^channels, since ano9 and ano10 inhibited anion conductance produced by ano1 [21]. So far no functional data exist for ano3 and ano4. Phylogenetic analysis suggests that anoctamin proteins descended from common ancestor and that ano8 and ano10 form a functional subfamily [20,25,26]. To gain more insight into the phylogeny and molecular evolution of the anoctamin gene family comprehensive bioinformatics study was performed. This has also led us to predict the structural and putative functional motifs, moreover a number of critical amino acid sites that may be of importance for the functional divergence in the anoctamin protein family have been identified.

## Results and discussion

### Origin and evolution of the anoctamin gene family

**Figure 1:**
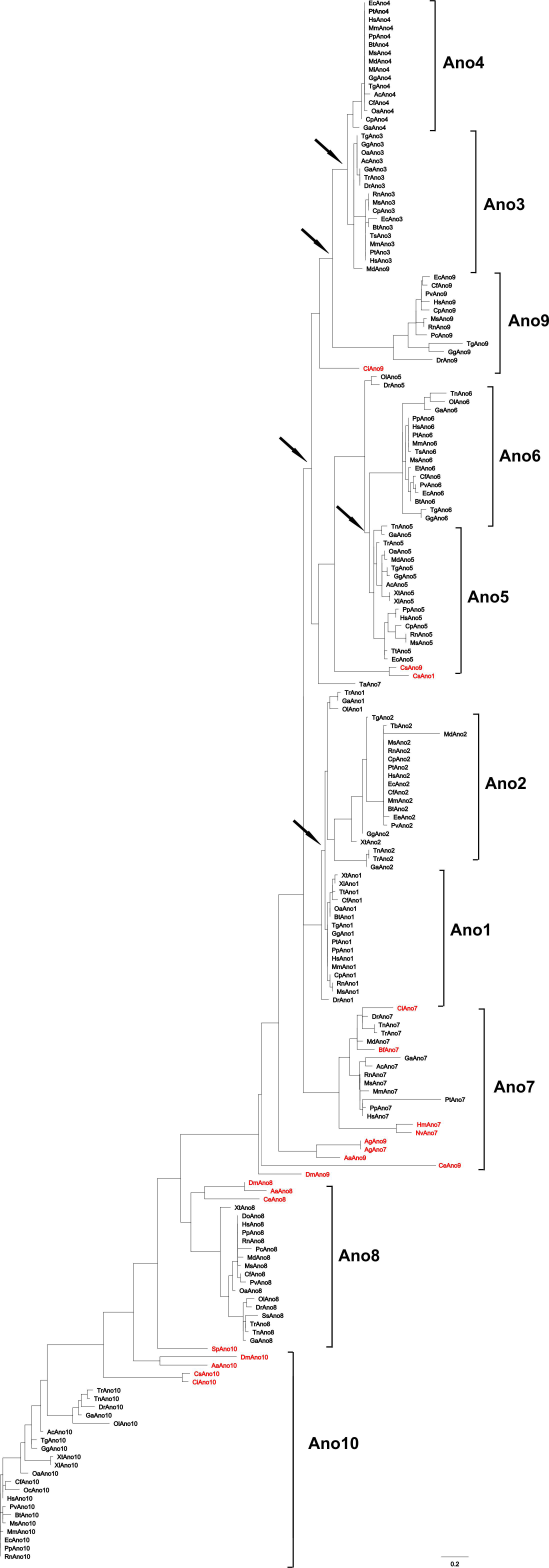
**Maximum likelihood tree of the anoctamin protein family**. The phylogenetic tree constructed with the program PhyML shows the evolutionary relationship of the anoctamin protein family. Several possible duplication time points are indicated with black arrows. Non-vertebrate anoctamins are depicted with red color. The unit of branch length is the expected fraction of amino acids substitution.

We first retrieved the available anoctamin sequences from the currently sequenced genomes. Querying major databases and unfinished genomes with the full-length amino acid sequences from the ten human anoctamin paralogues (ano1-10) identified 243 homologous proteins in vertebrates, urochordates, cephalochordates, echinodermates and invertebrates (Additional file 1). Incomplete and redundant sequences were discarded and initial data set included 186 sequences. To explore the phylogenetic relationship among anoctamin paralogues, we constructed an unrooted maximum-likelihood (ML) phylogenetic tree according to the best fit model (WAG+I+G) predicted using ProtTest program [27] for the 186 anoctamin genes from 50 species (Figure 1).

While vertebrates have 10 paralogs, most other organisms contain three or four anoctamin family members. Echinodermates (S. purpuratus) and the recently sequenced Amphioxus genome, which represents the best pre-duplicative set of the vertebrate genome [28] contains only one copy of the anoctamin gene, strongly suggesting that gene duplication events have occurred in the lineage leading to the vertebrates. In each of the urochordata genomes, Ciona inestinalis and Ciona savigny, the closest relatives of the craniates, we identified three anoctamin sequences. Thus, gene duplication of the anoctamin family appeared to have occurred very early at the base of the chordates tree. The vertebrate anoctamins form ten separate monophyletic groups, indicating that the formation of the paralogous subfamilies occurred before the divergence of individual species (Figure 1). The phylogenetic branches of anoctamins 8 and 10 separated considerably earlier in evolution than other anoctamin subgroups. The high level of sequence identity within a subfamily suggests evolutionarily conserved functions. Invertebrate genomes on the other hand contain distinctly fewer anoctamin paralogs, and it seems that their number increases with evolutionary complexity. Different number of anoctamin paralogs in invertebrates suggests complex evolutionary history. Overall, the data indicate that both, large scale (genome wide) and small-scale duplications contributed to the evolution of the anoctamin subfamilies, which is in good agreement with previous findings demonstrating that large-scale gene duplications have occurred during chordate evolution [29-31].

### Membrane topology of the vertebrate anoctamins

**Figure 2:**
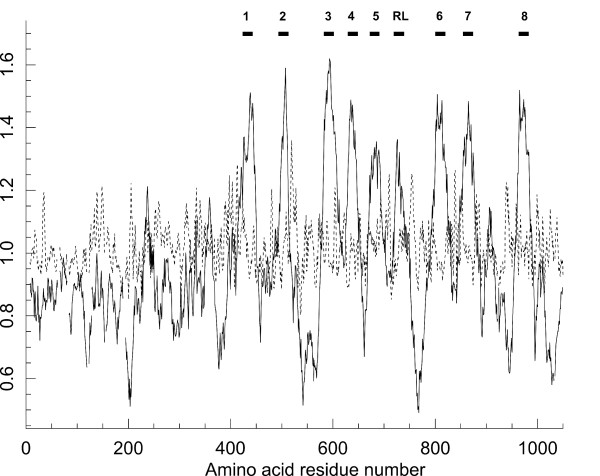
**Average hydropathy plot of 166 homologues of vertebrate bestrophins**. Hydropathy plot was generated from 166 vertebrate sequences as given in Additional file 1 using TMAP server which predicts transmembrane segments from an aligned set of proteins. Amino acid numbering corresponds to the numbers from the multiple sequence alignment. Black boxes depict predicted TMD's. RL = re-entry loop

For the analysis of the membrane topology we focused on vertebrate anoctamins. Multiple amino acid sequence alignment of 166 vertebrate anoctamins (Additional file 1) was used to predict putative transmembrane domains (TMDs) and hydrophobic regions. Hydropathy plotting of 166 anoctamins revealed eight transmembrane domains including one re-entry loop (Figure 2). These eight hydrophobic peaks are strongly conserved in the vertebrate anoctamins suggesting membrane insertion of all anoctamin family members, similar to anoctamin 1. These results are in agreement with topological study of ano7 [19].

### Evolution of the protein domains in the anoctamin family

**Figure 3:**
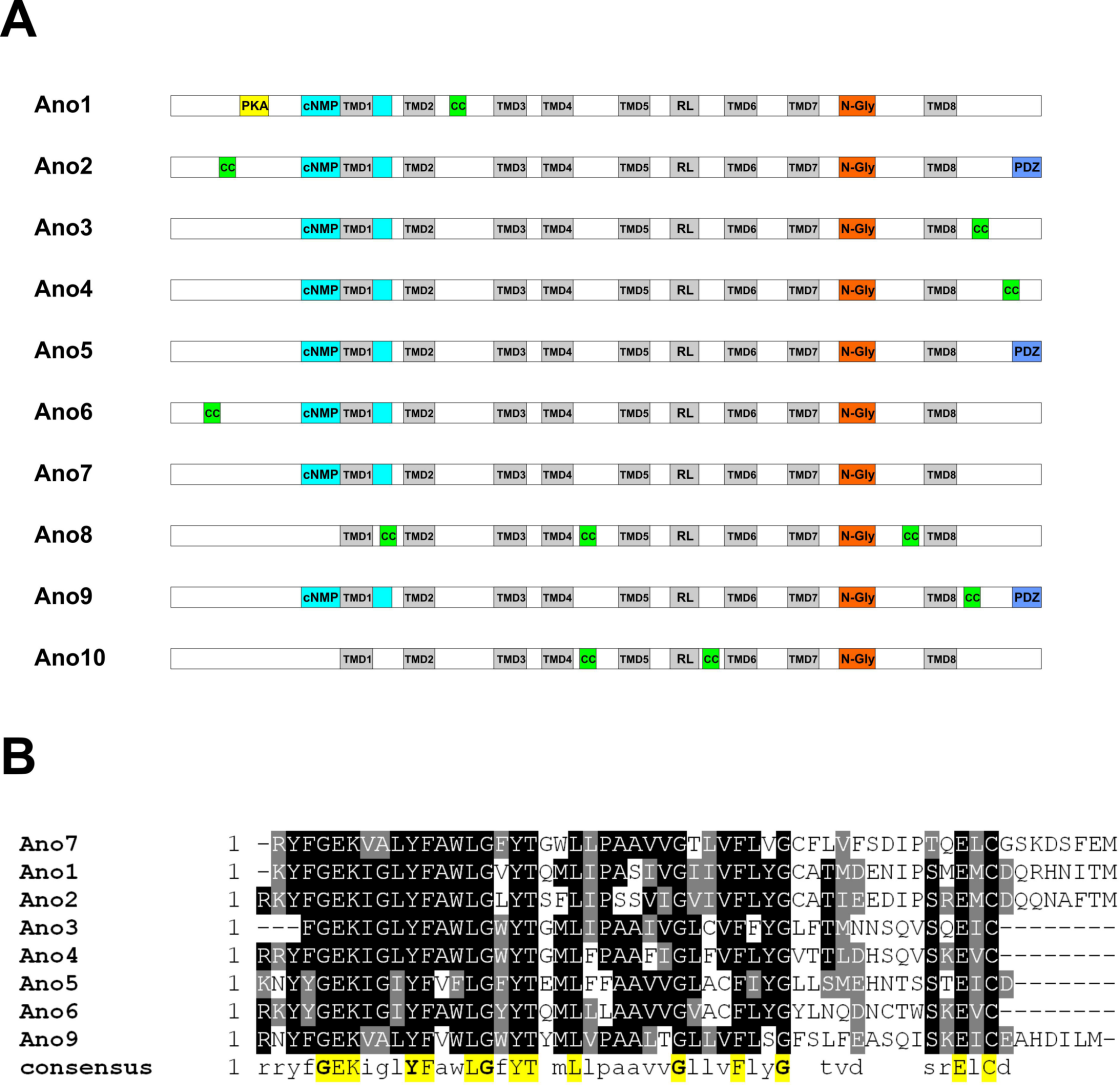
**Evolution of the protein domains in the anoctamin protein family**. **A**, Schematic representation of protein domains of anoctamin proteins in vertebrates. TMD, transmembrane domain; RL, re-entry loop; PDZ, PDZ domain, N-Gly, N-glycosilation site; PKA, protein kinase A phosphorylation site; cNMP, cyclic nucleotide-monophosphate binding domain. **B**, Amino acid sequence alignment of the representative anoctamin protein members containing putative cNMP binding site.

Anoctamin protein sequences were scanned for the presence of protein domains and functional sequence patterns with InterProScan and SMART servers (Figure 3). All anoctamins have at least one consensus N-glycosylation site, located in the extracellular loop between TMD7 and TMD8. Ano 2, 5 and 9 have a unique protein-protein interaction PDZ domains, although PDZ domain sequence in ano 5 and ano 9 is conserved only in mammals, and all anoctamins except ano 5 and ano 7 have at least one putative coiled-coil domain. A putative cyclic nucleotide-monophosphate binding domain (c-NMP) [32] which consists of a stretch of 60 amino acids containing α-helix and conserved amino acid residues located between TMD 1 and 2, is present in all anoctamins but ano 8 and 10 (Figure 3B). Thus, it appears that c-NMP binding domain evolved after splitting of ano 8 and 10 from the other anoctamin paralogs.

### Analysis of functional divergence

**Figure 4:**
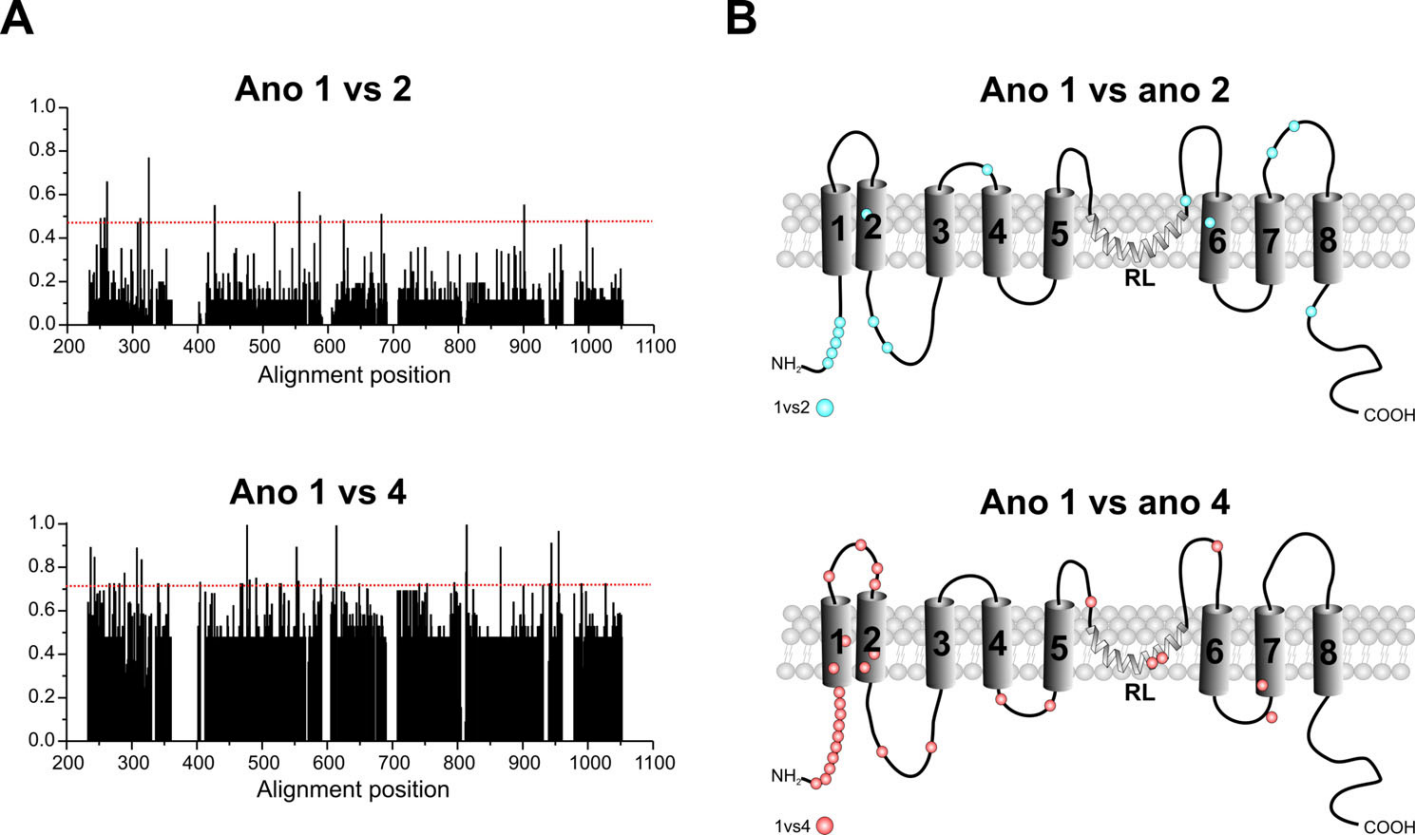
**Site specific profiles for evolutionary rate changes in the vertebrate anoctamin protein family**. **A**, The posterior probabilities of functional divergence for vertebrate anoctamins ano1, ano2 and ano4 were obtained with Diverge. Individual cut-off values for each comparison are marked with red horizontal lines. **B**, Residues with predicted functional divergence between anoctamin subfamilies are mapped onto the membrane topology model of ano 1.

Gene duplication provide a means to evolve novel biological functions and changes in protein functions may then provide different evolutionary constraints on duplicated genes. Functional divergence of a protein family can occur after major evolutionary events such as gene duplication or speciation. Some of them result in different evolutionary rates at certain amino acid residues, which is termed type I functional divergence [33,34]. To estimate functional divergence in the vertebrate anoctamin family, we have conducted pair-wise functional divergence analysis between anoctamin paralogous genes using DIVERGE [35]. Table 1 shows the coefficient of functional divergence (θ) of pair-wise comparisons between the members of the anoctamin family. All comparisons showed θ > 0 with p < 0.05, suggesting that a site-specific rate shift after gene duplication is a common phenomenon in the evolution of the anoctamin family. Further analysis was subsequently focused on ano1/ano2, and ano1/ano4. Amino acids residues responsible for functional divergence after gene duplication were identified using site-specific profiles (Figure 4A) in combination with suitable cut-off-values derived from the posterior probability of each comparison. Residues predicted to be functionally divergent in anoctamins were mapped onto topology model of human anoctamin 1 (Figure 4B). The predicted functional sites are not equally distributed throughout the respective anoctamin, but instead are clustered at the N-terminus, and in the hydrophilic loops between predicted transmembrane domains (Figure 4B). Despite the high global sequence identity of mammalian anoctamins 1 and 2, functionally divergent amino acids were also identified between these anoctamins. This amino acid residues which are predominantly located in the loop regions exposed to soluble ligands could be responsible for the different unitary conductance and kinetics between ano1 and ano 2 [1-3,6]. Finally, functional divergence within the anoctamin paralogues demonstrates that the anoctamin family members have evolved different functional properties after gene duplication and phylogenetic diversification events [4,6,21,22]. Although all anoctamins have similar membrane topology and show sequence conservation in the regions located around TMD's and the re-entrant loop, it is not clear whether all members of the anoctamin family are associated with Cl^- ^currents in various tissues. According to the result of our study, it is possible that they represent different types of ion channels, which can be activated by other types of physiological stimuli. This study also demonstrates that amino acids critical for functional divergence are predominantly located in the loop regions exposed to soluble ligands. Functional improvements which include pseudogene formation [36], subfunctionalization [37] and neofunctionlization [38] after gene duplication may result in altered functional constraints between members of a gene family. In this study, the divergences of amino acid sequences among different subfamilies provided us with indication that the anoctamin genes may have diverse physiological functions. The results of type I functional divergence (Table 1) suggested that anoctamin genes should be significantly functionally divergent from each other, owing to the evolutionary rate and/or property differences at some amino acid sites. Hence, functional divergence perhaps reflects the existence of long-term selective pressure.

**Table 1:** Estimates of the coefficient of functional divergence (θ)

	ano1/2	ano1/3	ano1/4	ano1/5	ano1/6	ano1/7	ano1/8	ano1/9	ano1/10	ano2/3	ano2/4	ano2/5	ano2/6	ano2/7	ano2/8
**ThetaML**	0.1448	0.3784	0.5176	0.332000	0.503333	0.145961	0.382400	0.446117	0.581600	0.5008	0.6768	0.662400	0.710400	0.608000	0.714400
**SE Theta**	0.048626	0.050346	0.066727	0.067265	0.075499	0.086110	0.074539	0.073320	0.060364	0.054403	0.081938	0.081977	0.092005	0.101139	0.087995
**alphaML**	0.526252	0.465416	0.51699	0.552795	0.510175	0.825007	0.666667	0.726183	0.491647	0.410835	0.448436	0.420455	0.364629	0.642576	0.458576
**LRT Theta**	8.867527	56.489153	60.170347	24.361073	44.445054	2.873167	26.318826	37.020919	92.830198	84.739938	68.225201	65.291364	59.618796	36.13849	65.912522

	**ano2/9**	**ano2/10**	**ano3/4**	**ano3/5**	**ano3/6**	**ano3/7**	**ano3/8**	**ano3/9**	**ano3/10**	**ano4/5**	**ano4/6**	**ano4/7**	**ano4/8**	**ano4/9**	**ano4/10**

**ThetaML**	0.722400	0.743200	0.428102	0.428000	0.510400	0.264800	0.521600	0.608800	0.776000	0.934400	0.796800	0.660800	0.801600	0.830556	0.999200
**SE Theta**	0.091732	0.072159	0.366065	0.082333	0.077775	0.091922	0.088668	0.076751	0.073366	0.124655	0.124385	0.135800	0.141002	0.130397	0.100264
**alphaML**	0.604621	0.372119	0.082326	0.418842	0.341202	0.646904	0.475797	0.576293	0.370614	0.522533	0.440092	0.830161	0.651255	0.717197	0.441753
**LRT Theta**	62.017573	106.078643	27.040848	27.023663	43.066508	8.298528	34.605454	62.9182	111.874879	56.188162	41.035693	23.677657	32.319444	40.569577	99.315745

	**ano5/6**	**ano5/7**	**ano5/8**	**ano5/9**	**ano5/10**	**ano6/7**	**ano6/8**	**ano6/9**	**ano6/10**	**ano7/8**	**ano7/9**	**ano7/10**	**ano8/9**	**ano8/10**	**ano9/10**

**ThetaML**	0.320000	0.308800	0.500000	0.485600	0.661600	0.414400	0.576000	0.479200	0.641600	0.386400	0.476987	0.547200	0.728800	0.605600	0.610400
**SE Theta**	0.063552	0.059126	0.064108	0.058921	0.049484	0.065456	0.070888	0.063839	0.053624	0.082841	0.061957	0.051296	0.069237	0.058657	0.049398
**alphaML**	0.468860	0.655629	0.545117	0.642576	0.458576	0.631854	0.506024	0.600512	0.415629	0.778094	0.782983	0.574307	0.696065	0.481043	0.568381
**LRT Theta**	25.353474	27.277167	60.830366	67.921915	178.758273	40.080804	66.023364	56.345669	143.158122	21.756284	59.268675	113.795056	110.799805	106.595188	152.687567

### Selective pressure among amino acid sites in the anoctamin family

**Figure 5:**
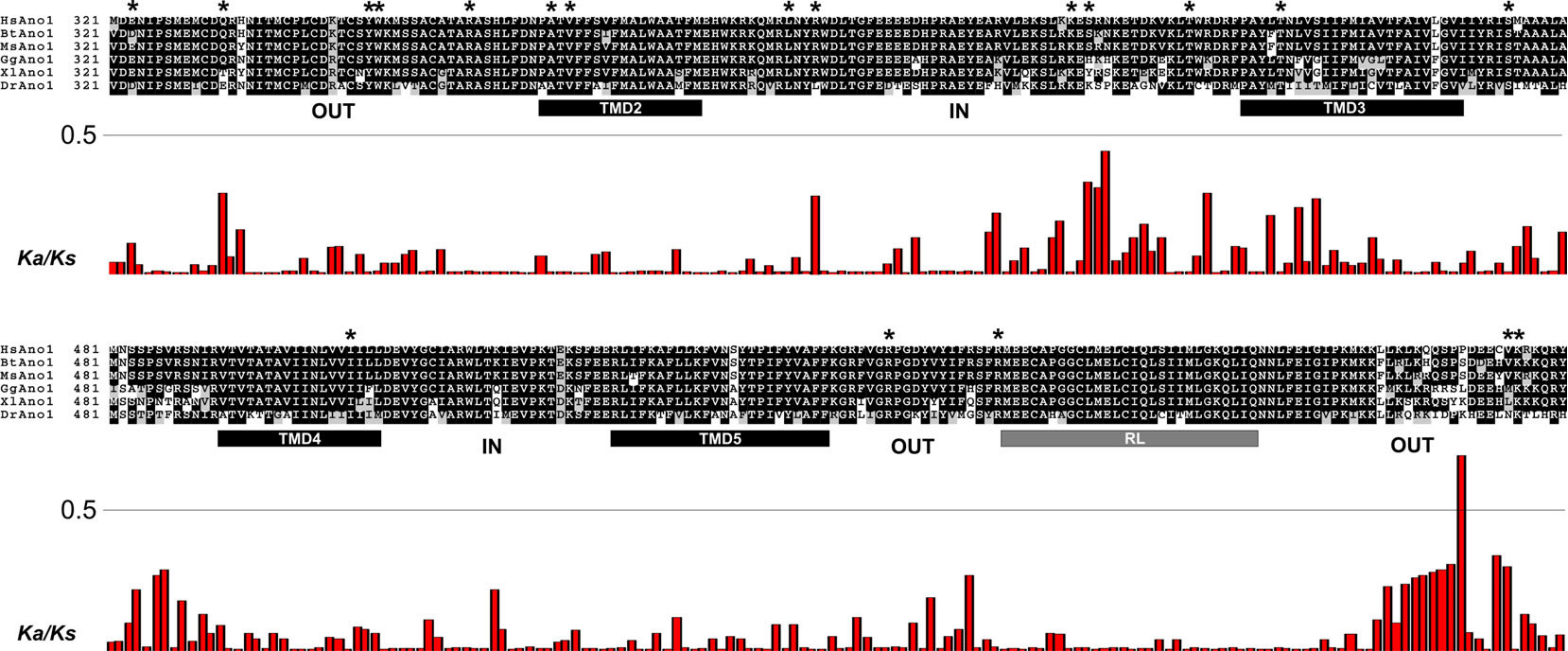
**Ka/Ks ratios and anoctamin 1 protein structure**. The results of Ka/Ks analysis on multiple alignment of ano1 proteins. Above the alignment, amino acids divergent between ano1, ano2 and ano4, are depicted with asterisk. Below the alignment is a histogram of the Ka/Ks ratios for each ungapped column of the alignment. IN/OUT indicates orientation with respect to the plasma membrane. Alignment shading indicates alignment quality.

In order to test for presence of positive selection at individual amino acid codons, the site specific models implemented in CODEML program [39] were used. Likelhood rate tests were performed between model M7 (beta) and M8 (beta and ω) on anoctamin sequences, however no positively selected sites were detected. This can be explained with strong purifying selection which acts on majority of the protein, while a few sites undergo positive selection. Therefore substitution rate ratios on non-synonymous (Ka) versus synonymous (Ks) mutations (Ka/Ks) were calculated for vertebrate anoctamins, as shown for anoctamin 1 (Figure 5). The ratios calculated between members of anoctamin family were much less then 1, such as 0.0741 for anoctamin 1, indicating strong negative selection. Interestingly, sites under strong purifying selection are located predominantly in the TMDs (Figure 5) suggesting their importance for the function of the anoctamin proteins.

## Conclusion

In conclusion, this comprehensive bioinformatics analysis of the anoctamin protein family suggests that both large-scale and small-scale gene duplications and purifying selection are the primary evolutionary force for generating the anoctamin family. Evolutionary analysis supports the hypothesis from electrophysiological studies that anoctamins have evolved distinctive functional properties, which have occurred after gene duplication(s). These findings will provide new insights for the structural evolution study of anoctamin gene family and possibly will offer a starting point for further experimental verifications.

## Methods

### Data collection and multiple sequence alignments

PSI-BLAST and TBLASTN [40] searches with protein sequences of the ten human anoctamins were performed in protein databases and available genome sequencing projects at NCBI, ENSEMBL, UniProt, InterPro, the Sanger Institute, UCSC Genome Bioinformatics Group, and the Joint Genome Institute. Proteins identified by the BLAST search algorithms were considered as potential homologues when amino acid identity was above 35% over a stretch of ≥150 amino acids. After removal of expressed sequence tags, alternatively spliced isoforms, partial and redundant sequences, the initial data set included 243 distinct sequences from 50 species (Additional file 1). Protein sequence alignments were performed using MUSCLE (Version 3.7) [41] and were subsequently manually edited to improve alignments in Bioedit. Sequences with highly divergent regions or gaps resulting in uncertain alignments were excluded from the further analysis. Remaining 186 sequences were subjected to MUSCLE alignments and subsequent phylogenetic analysis.

### Phylogenetic analysis

ProtTest v2.4 [27], implementing the Akaike Information criterion (AIC) was used to estimate the most appropriate model of amino acid substitution models for tree building analyses. The best fit model of protein evolution for the anoctamin protein family according to ProtTest corresponds to a JTT+I+G model [42]. Tree reconstructions were done by the Maximum Likelihood method (ML) from the protein alignment using PhyML software package [43], with the gamma distribution model implemented to account for heterogeneity among sites. The shape parameter of the gamma distribution (α) was estimated using baseml from the PAMLv4.0, to be α = 0.662. Support for each phylogenetic group was tested using 100 bootstrap pseudoreplicates.

### Topological analysis

Hydropathy analysis and prediction of putative transmembrane domains was done with the TMAP software [44], which is based on the Kyte and Doolittle algorithm. The average hydrophobicity values of putative transmembrane domains of 20-23 amino acid residues were calculated according the Eisenberg scale. An average hydropathy plot of 166 anoctamin-related protein sequences was generated by the TMAP software with a window of 19 amino acids.

### Functional divergence and detection of amino acids critical for altered functional constraints

Anoctamin sequence duplication events were tested for type I functional divergence based on the method by Gu et al [33,34]. The analysis was carried out with Diverge (version 2.0) [35]. This method is based on maximum likelihood procedures to estimate significant changes in the rate of evolution after the emergence of two paralogous sequences. Type I sites represent amino acid residues conserved in one subfamily but highly variable in another, implying that these residues have been subjected to different functional constraints. A set of 166 protein sequences was included in the study (Additional file 1, Supplemental Table S1). Due to of gaps in the alignment a total of 25 amino acid residues from human ano1 (codons 476-501), 61 (codons 1-61) from human ano2, 54 (codons 1-54) from human ano7, 33 (codons 749-782) of human ano9, and 46 (codons 1-28, and 639-660) from human ano10 were excluded from the analysis. A new NJ tree was constructed within Diverge with Poisson distance and re-rooted. The coefficient of functional divergence (θ) and the posterior probability for the functional divergence were calculated for each position in the alignment. To detect amino acid residues reflecting functional divergence, anoctamin subfamilies were pair-wise compared to each other. The cut-off value for the posterior probability was determined by consecutively eliminating the highest scoring residues from the alignment until the coefficient of functional divergence dropped to zero.

### Analysis of selective pressure

DNA sequences and related multiple proteins sequence alignments were submitted to the PAL2NAL web server [45] which converts a protein multiple sequence alignment and the corresponding DNA sequences into a codon alignment. Subsequently, the codon alignment and tree generated by using MUSCLE were provided to CODEML, and the site specific models M7 and M8 were tested.

## Authors' contributions

VMM conceived and carried out the study, performed statistical analyses, drafted the manuscript and prepared the figures. MB helped design the study, analyzing results and draft the manuscript. HS was involved in conceptual discussions for the entire study and made major revisions to the manuscript. BHFW and OS contributed to the study concept, corrected the manuscript and have given final approval of the version to be published. All authors read and approved the final manuscript.

## Supplementary Material

Additional file 1 **Anoctamin homologues (n = 243) used for phylogenetic analysis**. List of anoctamin homologues identified in public databases. This table lists the molecular features of all 243 anoctamin homologues identified in public databases. Click here for file
