# Supplementary material for: Evolution and functional divergence of the anoctamin family of membrane proteins
Source: BMC Evol Biol. 2010 Oct 21;10:319. doi: 10.1186/1471-2148-10-319 (PMC2974728; doi:10.1186/1471-2148-10-319)
Supplement: Additional file 1 — Anoctamin homologues (n = 243) used for phylogenetic analysis. List of anoctamin homologues identified in public databases. This table lists the molecular features of all 243 anoctamin homologues identified in public databases. [file 1471-2148-10-319-S1.PDF]

1	Name	Abr.	Taxonomy	Protein	aa	DNA	name	Common name	tree	topology
2	Bos taurus	Bt	Mammals	XP_610636	959	XM_610636	Ano1	cow	x	x
3	Canis familiaris	Cf	Mammals	XP_854489	1037	XM_849396	Ano1	dog	x	x
4	Cavia porcellus	Cp	Mammals	ENSCPOP00000009891	961	ENSCPOT00000011106	Ano1	Guinea Pig	x	x
5	Ciona savignyi	Cs	Urochordata	ENSCSAVP00000010779	859	ENSCSAVT00000010909	Ano1	sea squirt	x	
6	Danio rerio	Dr	Fish	XP_001923888	925	XM_001923853.1	Ano1	zebrafish	x	x
7	Equus caballus	Ec	Mammals	XP_001493686	845	XM_001493636	Ano1	horse		
8	Gallus gallus	Gg	Birds	XP_421072	955	XM_421072.2	Ano1	chicken	x	x
9	Gasterosteus aculeautus	Ga	Fish	ENSGACP00000003118	948	ENSGACT00000003130	Ano1	Three-spined stickleback	x	x
10	Homo sapiens	Hs	Mammals	NP_060513	986	NM_018043	Ano1	human	x	x
11	Macaca mulatta	Mm	Mammals	ENSMMUP00000025943	949	ENSMMUT00000027734	Ano1	macaca	x	x
12	Microcebus murinus	Mi	Mammals	ENSMICP00000015999	947	ENSMICT00000017568	Ano1	mouse lemur		
13	Monodelphis domestica	Md	Mammals	XP_001381079	1105	XM_001381042.1	Ano1	gray short-tailed opossum		
14	Mus musculus	Ms	Mammals	NP_848757	956	NM_178642	Ano1	mouse	x	x
15	Ornithorhynchus anatinus	Oa	Monotremata	XP_001507078	891	XM_001507028.1	Ano1	platypus	x	x
16	Oryzias latipes	OL	Fish	ENSORLP00000000347	891	ENSORLT00000000347	Ano1	Medaka	x	x
17	Pan troglodytes	Pt	Mammals	ENSPTRP00000006881	986	ENSPTRT00000007458	Ano1	chimpanzee	x	x
18	Pongo pygmaeus	Pp	Mammals	ENSPPYP00000003429	986	ENSPPYT00000003552	Ano1	orangutan	x	x
19	Rattus norvegicus	Rn	Mammals	NP_001101034	1040	NM_001107564	Ano1	rat	x	x
20	Taeniopygia guttata	Tg	Birds	ENSTGUP00000005601	952	ENSTGUT00000005659	Ano1	Zebra Finch	x	x
21	Takifugu rubripes	Tr	Fish	ENSTRUP00000044474	934	ENSTRUT00000044624	Ano1	fugu	x	x
22	Tetraodon nigroviridis	Tn	Fish	ENSTNIP00000007428	879	ENSTNIT00000007586	Ano1	Spotted Green Pufferfish		
23	Tursiops truncatus	Tt	Mammals	ENSTTRP00000001004	952	ENSTTRT00000001067	Ano1	bottlenosed dolphin	x	x
24	Xenopus laevis	Xl	Amphibia	NP_001128709	979	NM_001135237.1	Ano1	clawed frog	x	x
25	Xenopus tropicalis	Xt	Amphibia	NP_001123799	979	NM_001130327.1	Ano1	clawed frog	x	x
26	Aedes aegypti	Aa	Insects	AAEL010243-PA	792	AAEL010243-RA	Ano10	yellow fever mosquito	x	
27	Anolis carolinensis	Ac	Reptilia	ENSACAP00000014754	647	ENSACAT00000015055	Ano10	green anole	x	x
28	Anopheles gambiae	Ag	Insects	AGAP009776-PA	548	AGAP009776-RA	Ano10	Anopheles		
29	Bos taurus	Bt	Mammals	XP_614009	660	XM_614009	Ano10	cow	x	x
30	Canis familiaris	Cf	Mammals	XP_534209.2	659	XM_534209	Ano10	dog	x	x
31	Cavia porcellus	Cp	Mammals	ENSCPOP00000020316	601	ENSCPOT00000021343	Ano10	Guinea Pig		
32	Ciona intestinalis	Ci	Urochordata	ENSCINP00000007156	680	ENSCINT00000007396	Ano10	sea squirt	x	
33	Ciona savignyi	Cs	Urochordata	ENSCSAVP00000004603	663	ENSCSAVT00000004670	Ano10	sea squirt	x	
34	Danio rerio	Dr	Fish	NP_001025377.1	646	NM_001030206	Ano10	zebrafish	x	x
35	Drosophila melanogaster	Dm	Insects	FBpp0074146	646	FBtr0074372	Ano10	Fruitfly	x	
36	Equus caballus	Ec	Mammals	XP_001501420.1	660	XM_001501370.2	Ano10	horse	x	x
37	Gallus gallus	Gg	Birds	XP_418773.1	644	XM_418773.1	Ano10	chicken	x	x
38	Gasterosteus aculeautus	Ga	Fish	ENSGACP00000004996	658	ENSGACT00000005011	Ano10	Three-spined stickleback	x	x
39	Homo sapiens	Hs	Mammals	NP_060545.3	660	NM_018075	Ano10	human	x	x
40	Macaca mulatta	Mm	Mammals	XP_001114998.1	635	XM_001114998	Ano10	macaca	x	x
41	Monodelphis domestica	Md	Mammals	XP_001381303.1	1105	XM_001381266.1	Ano10	gray short-tailed opossum		
42	Mus musculus	Ms	Mammals	NP_598740.1	659	NM_133979.2	Ano10	mouse	x	x
43	Myotis lucifugus	Ml	Mammals	ENSMLUP00000007631	634	ENSMLUT00000008364	Ano10	Microbat		
44	Ornithorhynchus anatinus	Oa	Monotremata	XP_001509534.1	673	XM_001509484.1	Ano10	platypus	x	x
45	Oryctolagus cuniculus	Oc	Mammals	ENSOCUP00000007156	659	ENSOCUT00000008276	Ano10	rabbit	x	x
46	Oryzias latipes	Ol	Fish	ENSORLP00000004532	649	ENSORLT00000004533	Ano10	Medaka	x	x
47	Pan troglodytes	Pt	Mammals	XP_516396.2	678	XM_516396.2	Ano10	chimpanzee		
48	Pongo pygmaeus	Pp	Mammals	ENSPPYP00000015622	661	ENSPPYT00000016243	Ano10	orangutan	x	x
49	Procavia capensis	Pc	Mammals	ENSPCAP00000005900	661	ENSPCAT00000006313	Ano10	Hyrax		
50	Pteropus vampyrus	Pv	Mammals	ENSPVAP00000015317	662	ENSPVAT00000016237	Ano10	Megabat	x	x
51	Rattus norvegicus	Rn	Mammals	XP_236774.4	735	XM_236774.4	Ano10	rat	x	x
52	Strongylocentrotus purpuratus	Sp	Echinodermata	XP_786046.2	741	XM_780953.2	Ano10	purple sea urchin	x	
53	Taeniopygia guttata	Tg	Birds	ENSTGUP00000003952	649	ENSTGUT00000003994	Ano10	Zebra Finch	x	x
54	Takifugu rubripes	Tr	Fish	ENSTRUP00000033974	663	ENSTRUT00000034100	Ano10	fugu	x	x
55	Tetraodon nigroviridis	Tn	Fish	ENSTNIP00000000732	663	ENSTNIT00000001040	Ano10	Spotted Green Pufferfish	x	x
56	Vicugna pacos	Vp	Mammals	ENSVPAP00000005965	636	ENSVPAT00000006428	Ano10	Alpaca		
57	Xenopus laevis	Xl	Amphibia	AAH68693.1	624	BC068693	Ano10	clawed frog	x	x
58	Xenopus tropicalis	Xt	Amphibia	ENSXETP00000047668	606	ENSXETT00000047668	Ano10	clawed frog	x	x
59	Anolis carolinensis	Ac	Reptilia	ENSACAP00000009063	814	ENSACAT00000009256	Ano2	green anole		
60	Bos taurus	Bt	Mammals	XP_590066	1048	XM_590066.4	Ano2	cow	x	x
61	Canis familiaris	Cf	Mammals	ENSCAFP00000022508	993	ENSCAFT00000024244	Ano2	dog	x	x
62	Cavia porcellus	Cp	Mammals	ENSCPOP00000000819	914	ENSCPOT00000000915	Ano2	Guinea Pig	x	x
63	Danio rerio	Dr	Fish	ENSDARP00000087364	768	ENSDART00000092932	Ano2	zebrafish		
64	Equus caballus	Ec	Mammals	XP_001495378	1017	XM_001495328.2	Ano2	horse	x	x
65	Erinaceus europaeus	Ee	Mammals	ENSEEUP00000003425	991	ENSEEUT00000003758	Ano2	hedgehog	x	x
66	Gallus gallus	Gg	Birds	XP_425659	1051	XM_425659.2	Ano2	chicken	x	x
67	Gasterosteus aculeautus	Ga	Fish	ENSGACP00000017397	974	ENSGACT00000017431	Ano2	Three-spined stickleback	x	x
68	Homo sapiens	Hs	Mammals	NP_065106	998	NM_020373.2	Ano2	human	x	x
69	Macaca mulatta	Mm	Mammals	XP_001118212	1094	XM_001118212.1	Ano2	macaca		
70	Monodelphis domestica	Md	Mammals	XP_001368614	886	XM_001368577.1	Ano2	gray short-tailed opossum	x	x
71	Mus musculus	Ms	Mammals	NP_705817.2	1002	NM_153589.2	Ano2	mouse	x	x
72	Pan troglodytes	Pt	Mammals	XP_508944	999	XM_508944.2	Ano2	chimpanzee	x	x
73	Pongo pygmaeus	Pp	Mammals	ENSPPYP00000004762	933	ENSPPYT00000004951	Ano2	orangutan		
74	Pteropus vampyrus	Pv	Mammals	ENSPVAP00000015270	994	ENSPVAT00000016184	Ano2	Megabat	x	x
75	Rattus norvegicus	Rn	Mammals	XP_001066415	979	XM_001066415.1	Ano2	rat	x	x
76	Taeniopygia guttata	Tg	Birds	XP_002188249	1017	XM_002188213	Ano2	Zebra Finch	x	x
77	Takifugu rubripes	Tr	Fish	ENSTRUP00000009068	908	ENSTRUT00000009122	Ano2	fugu	x	x
78	Tetraodon nigroviridis	Tn	Fish	ENSTNIP00000000923	899	ENSTNIT00000003590	Ano2	Spotted Green Pufferfish	x	x
79	Tupaia belangeri	Tb	Mammals	ENSTBEP00000009431	988	ENSTBET00000010905	Ano2	tree shrew		
80	Xenopus tropicalis	Xt	Amphibia	ENSXETP00000018596	919	ENSXETT00000018596	Ano2	clawed frog	x	x
81	Anolis carolinensis	Ac	Reptilia	ENSACAP00000004530	988	ENSACAT00000004634	Ano3	green anole	x	x
82	Bos taurus	Bt	Mammals	XP_001789113	975	XM_001789061.1	Ano3	cow	x	x
83	Canis familiaris	Cf	Mammals	XP_534094	994	XM_534094.2	Ano3	dog		
84	Cavia porcellus	Cp	Mammals	ENSCPOP00000005023	982	ENSCPOT00000005636	Ano3	Guinea Pig	x	x
85	Danio rerio	Dr	Fish	XP_001922376	988	XM_001922341.1	Ano3	zebrafish	x	x
86	Dipodomys ordii	Do	Mammals	ENSDORP00000002624	965	ENSDORT00000002794	Ano3	Kangaroo rat		
87	Equus caballus	Ec	Mammals	XP_001502182	985	XM_001502132.2	Ano3	horse	x	x
88	Gallus gallus	Gg	Birds	XP_428890	1020	XM_428890.2	Ano3	chicken	x	x

89	Gasterosteus aculeautus	Ga	Fish	ENSGACP00000007843	985	ENSGACT00000007862	Ano3	Three-spined stickleback	x	x
90	Homo sapiens	Hs	Mammals	NP_113606	981	NM_031418.2	Ano3	human	x	x
91	Macaca mulatta	Mm	Mammals	XP_001091004	981	XM_001091004	Ano3	macaca	x	x
92	Monodelphis domestica	Md	Mammals	XP_001380157	890	XM_001380120.1	Ano3	gray short-tailed opossum	x	x
93	Mus musculus	Ms	Mammals	NP_001121575	981	NM_001128103.1	Ano3	mouse	x	x
94	Ornithorhynchus anatinus	Oa	Monotremata	XP_001509105.1	975	XM_001509055.1	Ano3	platypus	x	x
95	Oryzias latipes	Ol	Fish	ENSORLP00000007195	946	ENSORLT00000007196	Ano3	Medaka		
96	Pan troglodytes	Pt	Mammals	ENSPTRP00000006001	981	ENSPTRT00000006505	Ano3	chimpanzee	x	x
97	Pongo pygmaeus	Pp	Mammals	ENSPPYP00000003916	948	ENSPPYT00000004064	Ano3	orangutan		
98	Rattus norvegicus	Rn	Mammals	XP_001080134	965	XM_001080134.1	Ano3	rat	x	x
99	Taeniopygia guttata	Tg	Birds	ENSTGUP00000004825	980	ENSTGUT00000004874	Ano3	Zebra Finch	x	x
100	Takifugu rubripes	Tr	Fish	ENSTRUP00000030057	982	ENSTRUT00000030174	Ano3	fugu	x	x
101	Tarsius syrichta	Ts	Mammals	ENSTSYP00000011308	984	ENSTSYT00000012325	Ano3	Philippine Tarsier	x	x
102	Vicugna pacos	Vp	Mammals	ENSVPAP00000004813	981	ENSVPAT00000005188	Ano3	Alpaca		
103	Anolis carolinensis	Ac	Reptilia	ENSACAP00000012019	933	ENSACAT00000012264	Ano4	green anole	x	x
104	Bos taurus	Bt	Mammals	NP_001095520	920	NM_001102050	Ano4	cow	x	x
105	Canis familiaris	Cf	Mammals	ENSCAFP00000010300	955	ENSCAFT00000011119	Ano4	dog	x	x
106	Cavia porcellus	Cp	Mammals	ENSCPOP00000008054	981	ENSCPOT00000009045	Ano4	Guinea Pig	x	x
107	Danio rerio	Dr	Fish	ENSDARP00000088431	840	ENSDART00000097661	Ano4	zebrafish		
108	Equus caballus	Ec	Mammals	XP_001496953	955	XM_001496903	Ano4	horse	x	x
109	Gallus gallus	Gg	Birds	XP_425452.2	976	XM_425452	Ano4	chicken	x	x
110	Gasterosteus aculeautus	Ga	Fish	ENSGACP00000025786	920	ENSGACT00000025836	Ano4	Three-spined stickleback	x	x
111	Homo sapiens	Hs	Mammals	NP_849148	920	NM_178826	Ano4	human	x	x
112	Loxodonta africana	La	Mammals	ENSLAFP00000001810	965	ENSLAFT00000002165	Ano4	elephant		
113	Macaca mulatta	Mm	Mammals	XP_001090523	955	XM_001090523	Ano4	macaca	x	x
114	Microcebus murinus	Mi	Mammals	ENSMICP00000014307	920	ENSMICT00000015703	Ano4	mouse lemur	x	x
115	Monodelphis domestica	Md	Mammals	XP_001372398	1158	XM_001372361	Ano4	gray short-tailed opossum	x	x
116	Mus musculus	Ms	Mammals	ENSMUSP00000070528	955	ENSMUST00000070175	Ano4	mouse	x	x
117	Ornithorhynchus anatinus	Oa	Monotremata	ENSOANP00000008873	920	ENSOANT00000008875	Ano4	platypus	x	x
118	Pan troglodytes	Pt	Mammals	XP_509302	955	XM_509302	Ano4	chimpanzee	x	x
119	Pongo pygmaeus	Pp	Mammals	ENSPPYP00000005557	918	ENSPPYT00000005771	Ano4	orangutan	x	x
120	Rattus norvegicus	Rn	Mammals	ENSRNOP00000009084	782	ENSRNOT00000009084	Ano4	rat		
121	Taeniopygia guttata	Tg	Birds	ENSTGUP00000009305	917	ENSTGUT00000009404	Ano4	Zebra Finch	x	x
122	Anolis carolinensis	Ac	Reptilia	ENSACAP00000015027	841	ENSACAT00000015331	Ano5	green anole	x	x
123	Bos taurus	Bt	Mammals	XP_001787648.1	1245	XM_001787596.1	Ano5	cow		
124	Canis familiaris	Cf	Mammals	XP_542535.2	1371	XM_542535	Ano5	dog		
125	Cavia porcellus	Cp	Mammals	ENSCPOP00000012563	907	ENSCPOT00000014083	Ano5	Guinea Pig	x	x
126	Danio rerio	Dr	Fish	NP_001073452.1	900	NM_001079983	Ano5	zebrafish	x	x
127	Dipodomys ordii	Do	Mammals	ENSDORP00000004462	912	ENSDORT00000004769	Ano5	kangaroo rat		
128	Echinops telfairi	Et	Mammals	ENSETEP00000015411	911	ENSETET00000018978	Ano5	Lesser hedgehog tenrec		
129	Equus caballus	Ec	Mammals	XP_001918124.1	986	XM_001918089	Ano5	horse	x	x
130	Gallus gallus	Gg	Birds	ENSGALP00000005939	883	ENSGALT00000005949	Ano5	chicken	x	x
131	Gasterosteus aculeautus	Ga	Fish	ENSGACP00000007786	902	ENSGACT00000007805	Ano5	Three-spined stickleback	x	x
132	Homo sapiens	Hs	Mammals	NP_998764.1	913	NM_213599	Ano5	human	x	x
133	Monodelphis domestica	Md	Mammals	ENSMODP00000011594	858	ENSMODT00000011814	Ano5	gray short-tailed opossum	x	x
134	Mus musculus	Ms	Mammals	NP_808362.2	904	NM_177694	Ano5	mouse	x	x
135	Ornithorhynchus anatinus	Oa	Monotremata	ENSOANP00000020738	872	ENSOANT00000020741	Ano5	platypus	x	x
136	Oryzias latipes	Ol	Fish	ENSORLP00000007817	869	ENSORLT00000007818	Ano5	Medaka	x	x
137	Pan troglodytes	Pt	Mammals	ENSPTRP00000005995	844	ENSPTRT00000006499	Ano5	chimpanzee		
138	Pongo pygmaeus	Pp	Mammals	ENSPPYP00000003924	913	ENSPPYT00000004072	Ano5	orangutan	x	x
139	Pteropus vampyrus	Pv	Mammals	ENSPVAP00000004233	913	ENSPVAT00000004468	Ano5	Megabat		
140	Rattus norvegicus	Rn	Mammals	ENSRNOP00000049894	884	ENSRNOT00000042952	Ano5	rat	x	x
141	Taeniopygia guttata	Tg	Birds	ENSTGUP00000004695	870	ENSTGUT00000004744	Ano5	Zebra Finch	x	x
142	Takifugu rubripes	Tr	Fish	ENSTRUP00000031340	858	ENSTRUT00000031462	Ano5	fugu	x	x
143	Tetraodon nigroviridis	Tn	Fish	ENSTNIP00000016682	868	ENSTNIT00000016896	Ano5	Spotted Green Pufferfish	x	x
144	Tursiops truncatus	Tt	Mammals	ENSTTRP00000002366	912	ENSTTRT00000002523	Ano5	bottlenosed dolphin	x	x
145	Vicugna pacos	Vp	Mammals	ENSVPAP00000001285	883	ENSVPAT00000001391	Ano5	Alpaca		
146	Xenopus laevis	Xl	Amphibia	NP_001086810.1	896	NM_001093341.1	Ano5	clawed frog	x	x
147	Xenopus tropicalis	Xt	Amphibia	ENSXETP00000018385	854	ENSXETT00000018385	Ano5	clawed frog	x	x
148	Bos taurus	Bt	Mammals	XP_600052.3	911	XM_600052	Ano6	cow	x	x
149	Canis familiaris	Cf	Mammals	XP_852020.1	885	XM_846927.1	Ano6	dog	x	x
150	Choloepus hoffmanni	Ch	Mammals	ENSCHOP00000006037	909	ENSCHOT00000006835	Ano6	sloth		
151	Danio rerio	Dr	Fish	XP_001922463.1	861	XM_001922428.1	Ano6	zebrafish		
152	Echinops telfairi	Et	Mammals	ENSETEP00000009559	884	ENSETET00000011779	Ano6	lesser hedgehog tenrec	x	x
153	Equus caballus	Ec	Mammals	XP_001489574.2	1009	XM_001489524.2	Ano6	horse	x	x
154	Gallus gallus	Gg	Birds	XP_416045.2	912	XM_416045	Ano6	chicken	x	x
155	Gasterosteus aculeautus	Ga	Fish	ENSGACP00000024873	846	ENSGACT00000024922	Ano6	Three-spined stickleback	x	x
156	Homo sapiens	Hs	Mammals	NP_001020527.2	910	NM_001025356	Ano6	human	x	x
157	Macaca mulatta	Mm	Mammals	XP_001092876.1	910	XM_001092876.1	Ano6	macaca	x	x
158	Microcebus murinus	Mi	Mammals	ENSMICP00000004465	884	ENSMICT00000004898	Ano6	mouse lemur		
159	Mus musculus	Ms	Mammals	NP_780553.2	911	NM_175344	Ano6	mouse	x	x
160	Ochotona princeps	Op	Mammals	ENSOPRP00000001782	791	ENSOPRT00000001931	Ano6	American Pika		
161	Oryzias latipes	Ol	Fish	ENSORLP00000016875	860	ENSORLT00000016876	Ano6	Medaka	x	x
162	Otolemur garnettii	Og	Mammals	ENSOGAP00000013967	905	ENSOGAT00000015599	Ano6	Northern Greater Galago		
163	Pan troglodytes	Pt	Mammals	XP_509014.2	1130	XM_509014.2	Ano6	chimpanzee	x	x
164	Pongo pygmaeus	Pp	Mammals	ENSPPYP00000005059	860	ENSPPYT00000005257	Ano6	orangutan	x	x
165	Pteropus vampyrus	Pv	Mammals	ENSPVAP00000007004	907	ENSPVAT00000007422	Ano6	Megabat	x	x
166	Rattus norvegicus	Rn	Mammals	NP_001101578.1	799	NM_001108108	Ano6	rat		
167	Taeniopygia guttata	Tg	Birds	XP_002194483	894	XM_002194447	Ano6	Zebra Finch	x	x
168	Tarsius syrichta	Ts	Mammals	ENSTSYP00000012956	889	ENSTSYT00000014114	Ano6	Philippine tarsier	x	x
169	Tetraodon nigroviridis	Tn	Fish	ENSTNIP00000021655	862	ENSTNIT00000021890	Ano6	Spotted Green Pufferfish	x	x
170	Tursiops truncatus	Tt	Mammals	ENSTTRP00000001791	907	ENSTTRT00000001909	Ano6	bottlenosed dolphin		
171	Vicugna pacos	Vp	Mammals	ENSVPAP00000000435	887	ENSVPAT00000000465	Ano6	Alpaca		
172	Anolis carolinensis	Ac	Reptilia	ENSACAP00000001790	766	ENSACAT00000001834	Ano7	green anole	x	x
173	Anopheles gambiae	Ag	Insects	AGAP000095-PA	983	AGAP000095-RA	Ano7	Anopheles	x	
174	Bos taurus	Bt	Mammals	ENSBTAP00000040266	685	ENSBTAT00000042630	Ano7	cow		x
175	Branchiostoma floridae	Bf	Cephalochordata	XP_002204967.1	720	XM_002204931.1	Ano7	Amphioxus	x	
176	Canis familiaris	Cf	Mammals	XP_543329.2	1212	XM_543329.2	Ano7	dog		

177	Ciona intestinalis	Ci	Urochordata	XP_002125055.1	962	XM_002125019.1	Ano7	sea squirt	x	
178	Danio rerio	Dr	Fish	XP_001345787.2	893	XM_001345751.2	Ano7	zebrafish	x	x
179	Equus caballus	Ec	Mammals	ENSECAP00000005502	864	ENSECAT00000007520	Ano7	horse		
180	Gasterosteus aculeautus	Ga	Fish	ENSGACP00000005929	816	ENSGACT00000005946	Ano7	Three-spined stickleback	x	x
181	Homo sapiens	Hs	Mammals	NP_001001891.2	933	NM_001001891.3	Ano7	human	x	x
182	Macaca mulatta	Mm	Mammals	ENSMMUP00000002295	935	ENSMMUT00000002427	Ano7	macaca	x	x
183	Monodelphis domestica	Md	Mammals	XP_001377095.1	928	XM_001377058.1	Ano7	gray short-tailed opossum	x	x
184	Mus musculus	Ms	Mammals	NP_996914.1	859	NM_207031.1	Ano7	mouse	x	x
185	Nematostella vectensis	Nv	Cnidaria	XP_001641918.1	790	XM_001641868.1	Ano7	starlet sea anemone	x	
186	Ornithorhynchus anatinus	Oa	Monotremata	ENSOANP00000018644	775	ENSOANT00000018647	Ano7	platypus		
187	Pan troglodytes	Pt	Mammals	XP_001161327.1	918	XM_001161327.1	Ano7	chimpanzee	x	x
188	Pongo pygmaeus	Pp	Mammals	ENSPPYP00000014935	843	ENSPPYT00000015533	Ano7	orangutan	x	x
189	Rattus norvegicus	Rn	Mammals	NP_001004071.1	860	NM_001004071.1	Ano7	rat	x	x
190	Takifugu rubripes	Tr	Fish	ENSTRUP00000011336	806	ENSTRUT00000011396	Ano7	fugu	x	x
191	Tetraodon nigroviridis	Tn	Fish	ENSTNIP00000013829	837	ENSTNIT00000014024	Ano7	Spotted Green Pufferfish	x	x
192	Trichoplax adhaerens	Ta	Placozoa	XP_002113853	727	XM_002113817	Ano7	Trichoplax	x	
193	Trichoplax adhaerens	Ta	Placozoa	XP_002110883.1	900	XM_002110847	Ano7	Trichoplax	x	
194	Aedes aegypti	Aa	Insects	AAEL004982-PA	800	AAEL004982-RA	Ano8	yellow fever mosquito	x	
195	Bos taurus	Bt	Mammals	XP_001790019.1	1146	XM_001789967.1	Ano8	cow		
196	Caenorhabditis elegans	Ce	Nematode	Y57G11C.37	837	Y57G11C.37	Ano8	roundworm	x	
197	Canis familiaris	Cf	Mammals	XP_541955.2	1107	XM_541955.2	Ano8	dog	x	x
198	Danio rerio	Dr	Fish	XP_691248.3	1120	XM_686156.3	Ano8	zebrafish	x	x
199	Dipodomys ordii	Do	Mammals	ENSDORP00000011983	877	ENSDORT00000012741	Ano8	Kangaroo rat	x	x
200	Drosophila melanogaster	Dm	Insects	FBpp0089099	1338	FBtr0080703	Ano8	Fruitfly	x	
201	Equus caballus	Ec	Mammals	ENSECAP00000012042	1064	ENSECAT00000015020	Ano8	horse		
202	Gasterosteus aculeautus	Ga	Fish	ENSGACP00000010349	1105	ENSGACT00000010371	Ano8	Three-spined stickleback	x	x
203	Homo sapiens	Hs	Mammals	NP_066010.1	1232	NM_020959.1	Ano8	human	x	x
204	Macaca mulatta	Mm	Mammals	ENSMMUP00000021332	1217	ENSMMUT00000022806	Ano8	macaca		
205	Monodelphis domestica	Md	Mammals	ENSMODP00000018868	1219	ENSMODT00000019208	Ano8	gray short-tailed opossum	x	x
206	Mus musculus	Ms	Mammals	XP_894573.1	1060	XM_889480.2	Ano8	mouse	x	x
207	Ochotona princeps	Op	Mammals	ENSOPRP00000005267	1219	ENSOPRT00000005736	Ano8	American Pika		
208	Ornithorhynchus anatinus	Oa	Monotremata	XP_001518745.1	1242	XM_001518695.1	Ano8	platypus	x	x
209	Oryzias latipes	Ol	Fish	ENSORLP00000011107	1054	ENSORLT00000011108	Ano8	Medaka	x	x
210	Pan troglodytes	Pt	Mammals	ENSPTRP00000018205	1043	ENSPTRT00000019674	Ano8	chimpanzee		
211	Pongo pygmaeus	Pp	Mammals	ENSPPYP00000010879	1010	ENSPPYT00000011303	Ano8	orangutan	x	x
212	Procavia capensis	Pc	Mammals	ENSPCAP00000009603	1138	ENSPCAT00000010290	Ano8	Hyrax	x	x
213	Pteropus vampyrus	Pv	Mammals	ENSPVAP00000007434	1229	ENSPVAT00000007876	Ano8	Megabat	x	x
214	Rattus norvegicus	Rn	Mammals		950	ENSRNOT00000038524	Ano8	rat	x	x
215	Salmo salar	Ss	Fish	ACN10494.1	1049	BT058781	Ano8	Atlantic salmon	x	x
216	Takifugu rubripes	Tr	Fish	ENSTRUP00000008104	1064	ENSTRUT00000008152	Ano8	fugu	x	x
217	Tetraodon nigroviridis	Tn	Fish	CAF91064.1	1068	CAAE01008023.1	Ano8	Spotted Green Pufferfish	x	x
218	Xenopus tropicalis	Xt	Amphibia	ENSXETP00000028651	1021	ENSXETT00000028651	Ano8	clawed frog	x	x
219	Aedes aegypti	Aa	Insects	AAEL006656-PA	1014	AAEL006656-RA	Ano9	yellow fever mosquito	x	
220	Anolis carolinensis	Ac	Reptilia	ENSACAP00000013317	568	ENSACAT00000013587	Ano9	green anole		
221	Anopheles gambiae	Ag	Insects	AGAP000095-PA	983	AGAP000095-RA	Ano9	Anopheles	x	
222	Bos taurus	Bt	Mammals	XP_589879.3	531	XM_589879.4	Ano9	cow		
223	Caenorhabditis elegans	Ce	Nematode	F56A8.1	1049	F56A8.1	Ano9	roundworm	x	
224	Canis familiaris	Cf	Mammals	XP_540519	734	XM_540519.2	Ano9	dog	x	x
225	Cavia porcellus	Cp	Mammals	ENSCPOP00000003330	772	ENSCPOT00000003734	Ano9	Guinea Pig	x	x
226	Ciona intestinalis	Ci	Urochordata	ENSCINP00000011437	792	ENSCINT00000011437	Ano9	sea squirt	x	
227	Ciona savignyi	Cs	Urochordata	ENSCSAVP00000010778	857	ENSCSAVT00000010908	Ano9	sea squirt	x	
228	Danio rerio	Dr	Fish	XP_001922660.1	788	XM_001922625.1	Ano9	zebrafish	x	x
229	Drosophila melanogaster	Dm	Insects	FBpp0112943	984	FBtr0114451	Ano9	Fruitfly	x	
230	Echinops telfairi	Et	Mammals	ENSETEP00000003530	531	ENSETET00000004312	Ano9	lesser hedgehog tenrec		
231	Equus caballus	Ec	Mammals	XP_001489165.1	743	XM_001489115.1	Ano9	horse	x	x
232	Gallus gallus	Gg	Birds	XP_420928.2	794	XM_420928.2	Ano9	chicken	x	x
233	Gasterosteus aculeautus	Ga	Fish	ENSGACP00000018642	629	ENSGACT00000018679	Ano9	Three-spined stickleback		
234	Homo sapiens	Hs	Mammals	NP_001012302.2	782	NM_001012302	Ano9	human	x	x
235	Macaca mulatta	Mm	Mammals	ENSMMUP00000037878	737	ENSMMUT00000044856	Ano9	macaca		
236	Monodelphis domestica	Md	Mammals	XP_001380157.1	890	XM_001380120.1	Ano9	gray short-tailed opossum	x	x
237	Mus musculus	Ms	Mammals	NP_848468.2	747	NM_178381.3	Ano9	mouse	x	x
238	Oryzias latipes	Ol	Fish	ENSORLP00000007382	549	ENSORLT00000007383	Ano9	Medaka		
239	Procavia capensis	Pc	Mammals	ENSPCAP00000012994	745	ENSPCAT00000013902	Ano9	Hyrax	x	x
240	Pteropus vampyrus	Pv	Mammals	ENSPVAP00000013246	773	ENSPVAT00000014046	Ano9	Megabat	x	x
241	Rattus norvegicus	Rn	Mammals	XP_574586.2	765	XM_574586.2	Ano9	rat	x	x
242	Taeniopygia guttata	Tg	Birds	ENSTGUP00000006956	714	ENSTGUT00000007025	Ano9	Zebra Finch	x	x
243	Takifugu rubripes	Tr	Fish	ENSTRUP00000032372	628	ENSTRUT00000032496	Ano9	fugu		x
244	Tetraodon nigroviridis	Tn	Fish	ENSTNIP00000009167	548	ENSTNIT00000009338	Ano9	Spotted Green Pufferfish		x
